# Reliability and validity of the Activity Questionnaire for Adults and Adolescents (AQuAA)

**DOI:** 10.1186/1471-2288-9-58

**Published:** 2009-08-10

**Authors:** Mai JM Chinapaw, Sander M Slootmaker, Albertine J Schuit, Mariska van Zuidam, Willem van Mechelen

**Affiliations:** 1 EMGO-Institute for Health and Care research, Department of Public and Occupational Health, VU University Medical Center, van der Boechorststraat 7, 1081 BT Amsterdam, The Netherlands; 2 Division of Public Health and Health Care, National Institute for Public Health and the Environment, PO Box 1, 3720 B Bilthoven, The Netherlands

## Abstract

**Background:**

Accurate measures of physical activity are highly needed. We evaluated the test-retest reliability and construct validity of the self-report Activity Questionnaire for Adults and Adolescents (AQuAA). The AQuAA is a commonly used questionnaire in Dutch youth.

**Methods:**

In the test-retest reliability study, 53 adolescents and 58 adults completed the AQuAA twice, with an interval of two weeks. In the validity study, 33 adolescents and 47 adults wore an accelerometer (Actigraph) during two weeks, and subsequently completed the AQuAA.

**Results:**

In adolescents the test-retest reliability was fair to moderate (intraclass correlations (ICCs) ranging from 0.30 to 0.59). In adults the test-retest reliability was fair to moderate for the time spent on sedentary, light and moderate intensity activities (ICCs ranging from 0.49 to 0.60), but poor for time spent on vigorous activities (ICC = -0.005). The correlations between the AQuAA and Actigraph were low and nonsignificant. Compared with the Actigraph, time spent on all physical activities was significantly higher according to the questionnaire (except for light intensity activities in adolescents), while time spent on sedentary behaviours was significantly lower.

**Conclusion:**

Reliability of the AQuAA is fair to moderate. The validity of the AQuAA compared to an accelerometer is poor. Both adolescents and adults underestimate the time spent on sedentary behaviours and overestimate the time spent on physical activities.

## Background

Physical activity is an important behaviour related to a number of health outcomes. Accurate assessment of physical activity levels is important to understand the association between physical activity and health, but also to monitor secular trends in behaviour and to evaluate the effectiveness of interventions and programs [1]. However, valid and appropriate assessment of physical activity (PA) is a challenging task. First, since PA behaviour varies considerably within and among individuals and populations. Second, there are several health-related dimensions of PA, such as caloric expenditure, aerobic intensity, weight bearing, flexibility, and strength [2,3].

Epidemiological studies have typically used subjective measures, such as the questionnaire, to assess PA in populations. PA questionnaires are easy to administer, non-reactive (does not alter the behaviour of the individual being surveyed), relatively inexpensive and accepted by study participants [2,4]. Dependent on the research question a different type of information is needed, e.g. sports activities, leisure time activities, work-related activities and active transportation. In addition, interest can be on 'habitual' or usual PA or PA in the past day(s), week, month, year or even a lifetime [2]. Hence, many questionnaires have been developed for different purposes. Few of these questionnaires also focus on sedentary behaviour. Independent of PA, sedentary behaviour is associated with obesity, a risk factor for many chronic diseases [5]. Therefore, it is important to assess the amount of time spent on specific sedentary behaviours such as watching TV and computer use as well.

PA questionnaires are usually developed for specific age groups. A disadvantage of age-group specific questionnaires is that levels of PA are difficult to compare. To be able to compare PA levels between different age groups, one questionnaire that can be used in different age groups and which estimates PA in a standardized way would be valuable. Therefore, we developed the **A**ctivity **Qu**estionnaire for **A**dolescents and **A**dults (AQuAA), a questionnaire with an adult as well as an adolescent version providing the same information about physical activity in both age groups.

The aim of the **A**ctivity **Qu**estionnaire for **A**dolescents and **A**dults (AQuAA) is to estimate light, moderate, vigorous, and total PA, but also sedentary behaviour among both adolescents and adults. The AQuAA instrument is nowadays commonly used in the Netherlands e.g. for monitoring national trends in physical activity among youth or evaluating interventions [6-8]. However, up to know now there is no data on its validity and reliability.

This paper presents two studies: one study investigating the test-retest reliability, and another study investigating construct validity of the AQuAA compared to an accelerometer. Both studies were performed among adolescents and adults between 12 and 38 years of age.

## Methods

### The Activity Questionnaire for Adolescents and Adults

The structure of the AQuAA is based on a previously developed Dutch physical activity questionnaire for adults (SQUASH [9]). The SQUASH was not designed to measure energy expenditure, but to give an indication of the habitual activity level. The SQUASH was structured in such a way that it would be possible to assess compliance to Dutch physical activity guidelines i.e. at least moderate intensity PA for a minimum of 30 minutes on at least five days of the week for adults and at least moderate intensity PA with a minimum of one hour a day for adolescents)[10]. The choice of activities included in the SQUASH was based on their intensity (≥ 4 MET). Thus, the SQUASH does not include questions on light intensity PA or sedentary behaviours except for light household activities and light activities at work and school. The AQuAA was developed for evaluation of the effectiveness of an intervention aimed at promoting physical activity for adolescents and young adults. We modified the SQUASH since we needed a questionnaire that 1) measures both physical activity as well as sedentary behaviour; 2) can be self-completed by adolescents as well as young adults; 3) is suitable for assessing changes over short periods of time. For this specific purpose we made the following adaptations: 1) The AQuAA contains questions on light, moderate and vigorous intensity activities as well as sedentary behaviours; 2) To improve the validity of the answers we included age-specific examples of activities; 3) The questions in the AQuAA relate to activities performed in the previous seven days, instead of 'an average week in the past months'. We decided to recall the past 7 days because with short time frames the estimates are less vulnerable to recall bias and more practical to validate with objective tools [2].

Physical activities are divided in five categories: i.e. commuting activities; physical activities at work or school; household activities; leisure time activities; and active sports [see Additional file 1]. Each category includes questions on time spent on various activities with examples of activities to facilitate completion. The only difference between the questionnaire for adults and adolescents are the examples provided. For each activity the frequency ('how many days in the past week'), duration ('how long') and perceived intensity ('low', 'medium' or 'high') are asked for. Completion of the questionnaire takes on average 15 to 20 minutes.

The five main outcomes are a total physical activity score (AQuAA score including all activities above 2 MET in MET*min/wk), and time spent on sedentary, light, moderate and vigorous intensity activities in minutes per week. Table 1 presents the cut-off values for sedentary, light, moderate and vigorous intensity activity. The AQuAA score is based on the sum of all activities ≥ 2 MET, expressed in minutes per week times the corresponding MET-value according to Ainsworth (i.e. MET*min/wk)[11]. Thus if a person reports to have walked twice a week at light intensity (2.5 MET) for 1 hour and 30 minutes the calculation would be as follows:

**Table 1:** Cut-off values for light, moderate and vigorous physical activities for adolescents and adults [10].

	Adolescents (≤ 18 yr)		Adults (18–55 yr)	
**Activity intensity**	**MET Range**	**Accelerometer counts (counts per minute)**	**MET Range**	**Accelerometer counts (counts per minute)**

**Sedentary**	< 2	< 699	< 2	< 699
**Light**	2 – 5	700 – 4478	2 – 4	700 – 3220
**Moderate**	5 – 8	4479 – 8252	4 – 6.5	3221 – 6365
**Vigorous**	≥ 8	≥ 8253	≥ 6.5	≥ 6366

→ 2 × 90 min = 180 min/week

→ 2.5 MET = labelled as light intensity for both adults/adolescents

→ 180*2.5 = 450 MET*min/week

### Procedures

The reliability and validity study were conducted in April 2005, analysed separately and involved different subjects. The Medical Ethics Committee of the VU University Medical Center approved the study protocol.

### Test-retest reliability study

**Figure 1:**
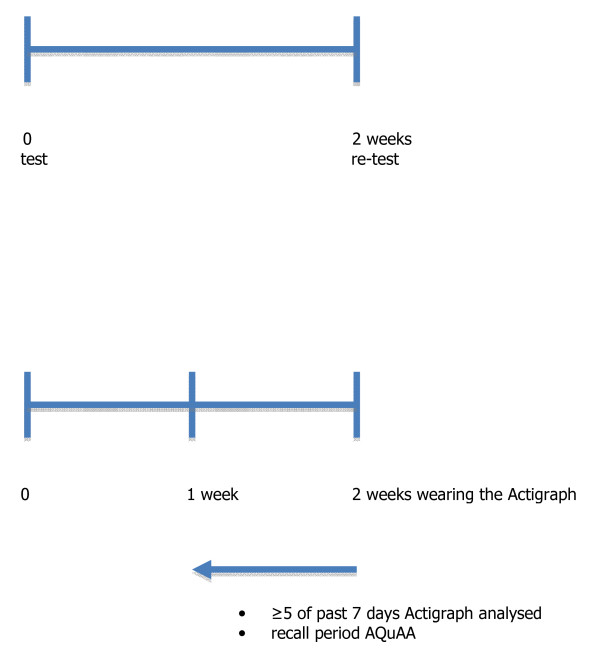
**Timing of the measurements in the reliability and validity studies**. a. The timing of the measurements in the reliability study. b. The timing of the measurements in the validity study.

A sample of adolescents (n = 59) aged 12 to 16 was recruited from a high school in a middle-sized city in the Netherlands. A sample of young adults (n = 63) aged 25 to 38 was recruited from a soccer team, a department of the KLM Royal Dutch Airlines and a department of the VU University Medical Center in Amsterdam. Both samples administered the questionnaire twice with an interval of two weeks (see figure 1). Adolescents filled in the questionnaire in the classroom with classmates supervised by the teacher and a research assistant. Adults filled in the questionnaire within the particular setting supervised by a research assistant. The research assistant checked the questionnaires when they were returned. Subjects who were not available on both measurement occasions were excluded (six adolescents and five young adults). All subjects (and the parents of the adolescents) signed an informed consent.

### Data analysis

The test-retest reliability of the AQuAA questionnaire was determined by comparing the results on the two separate occasions the questionnaire was filled in. The Intra Class Correlation coefficient (ICC) was calculated for the AQuAA score, and for the amount of time spent on sedentary, light, moderate and vigorous intensity activities, respectively. This was done for adolescents and young adults separately. An ICC < 0.40 was rated as poor agreement, 0.40–0.75 as fair to good agreement, and values > 0.75 as excellent agreement [12].

### Validity study

The construct validity of the AQuAA was assessed by comparison with the Actigraph accelerometer model 7164 (MTI, USA). The Actigraph is small (5 × 5 × 1.5 cm), lightweight (56.7 g) and can be worn on the wrist, ankle or hip. In the present study the Actigraph was worn on the right hip attached to a belt. Although the Actigraph underestimates some activities, such as cycling and water activities, it is recognised as a reasonably valid tool to assess PA objectively in adolescents [13,14] as well as adults [15].

A sample of 65 adolescents was recruited from four high schools in the Netherlands. A sample of 56 young adults was recruited from three different companies in the Netherlands (McDonalds headquarters, Solvay Pharmaceuticals and NS Dutch Railways). All subjects (and the parents of the adolescents) signed an informed consent. Adolescents filled in the questionnaire in the classroom with classmates supervised by the teacher and a research assistant. Adults filled in the questionnaire at their worksite supervised by a research assistant. The research assistant checked the questionnaires when they were returned.

All included participants received instruction to wear the Actigraph during two weeks from the time they woke up until they went to sleep, and to take it off when taking a shower or during watersport participation. The Actigraph was set to collect acceleration data with an interval of one minute. At the end of the two weeks all participants returned their Actigraph and immediately filled in the AQuAA. Vertical acceleration measures of the Actigraph were converted into activity counts per minute. With the regression equation of Freedson et al. [16] these counts were categorized as light, moderate or vigorous intensity PA based on age-specific cut-off points. Subsequently, minutes spent on sedentary, light, moderate and vigorous PA were calculated. The total Actigraph score was determined by dividing the total counts during at least light intensity activities of the Actigraph by the number of minutes a person had worn the Actigraph. Data of participants who wore the Actigraph at least 12 hours per day (i.e. 75% of the waking hours) and for at least five of the seven days that were recalled by the AQuAA were used in the analysis (see figure 1). Data of 23 adolescents and nine adults were excluded from analysis because of incomplete accelerometer data. Most of these subjects forgot or refused to wear the Actigraph. For two of these subjects (one adolescent and one adult) the Actigraph did not properly register the data. Finally, 42 adolescents (21 male, 21 female) and 47 young adults (20 male, 28 female) were included in the analysis.

### Data analysis

The following hypotheses were tested to assess construct validity:

1. Previous studies validating self-administered questionnaires recalling 7 days against accelerometry found correlations between -0.26 and 0.40 in youth and between 0.23 and 0.36 in adults [17,18]. We expected the correlation between the time (min/wk) spent on sedentary, light, moderate and vigorous intensity activities according to the AQuAA and Actigraph would be at least 0.30 in adolescents as well as adults.

2. The correlation between time spent on vigorous activity according to the AQuAA and the Actigraph will be higher than the correlation between time spent on moderate or light intensity activities according to the AQUAA and Actigraph.

Since the scores were not distributed normally we computed Spearman correlation coefficients to test hypotheses 1 and 2.

In addition, to assess the group level-validity of the AQuAA the median difference (and 25^th^–75^th ^percentile) for time spent on sedentary, light, moderate and vigorous intensity activities between the Actigraph and the questionnaire we subtracted the minutes according to the Actigraph by the minutes according to the questionnaire, for the respective categories. This was done both for adolescents and for young adults.

For validity studies as a rule of thumb, a sample size of at least 50 subjects is considered adequate, based on a general guideline by Altman [19]. Probability (p) values less than 0.05 were considered significant. All analyses were done using the Statistical Package of Social Sciences, version 11.1 for Windows (SPSS Inc, Chicago, Illinois, USA).

## Results

### Test-retest reliability study

The test-retest reliability study included 53 adolescents (30 male, 23 female with a mean age of 14.1 ± 1.4 yrs) and 58 young adults (20 male, 28 female with a mean age of 28.9 ± 3.5 yrs). Table 2 shows the AQuAA score, minutes per week spent on sedentary behaviours, light, moderate and vigorous intensity PA at test one and test two, and the corresponding ICCs. For adolescents the ICCs for the AQuAA score, sedentary behaviours, moderate and vigorous intensity PA ranged from 0.44 to 0.59, representing fair to moderate agreement. For the time spent on light PA the agreement between the two tests was poor. For adults the ICCs for time spent on sedentary behaviours, light and moderate intensity PA ranged from 0.49 to 0.60, representing fair to moderate agreement. For the AQuAA score and time spent on vigorous PA the agreement between the two tests was poor.

**Table 2:** Test-retest correlations (ICCs) of the AQuAA scores for adolescents and adults.

	Adolescents (N = 53)^a^			Adults (n = 58)^b^		
	**T1** **Median** **(25–75th percentile)**	**T2** **Median** **(25–75th percentile)**	*ICC* **(95% CI)**	**T1** **Median** **(25–75th percentile)**	**T2** **Median** **(25–75th percentile)**	**ICC** **(95% CI)**

AQuAA score (MET*min/wk)	17980 (12801;29519)	13982 (9668;21115)	0.44 (0.16;0.65)	11992 (9476;14966)	11395 (9731;140323)	0.22 (-0.04;0.46)
Sedentary activities (min/wk)	3750 (2858;4965)	3130 (2460;4170)	0.57 (0.34;0.73)	2930 (2220;3893)	2880 (2370;3678)	0.60 (0.40;0.74)
light activities (min/wk)	1250 (578;2115)	600 (300;1358)	0.30 (0.04;0.52)	1140 (554;1450)	728 (454;1376)	0.49 (0.27;0.66)
moderate activities (min/wk)	540 (295;1268)	470 (195;1358)	0.50 (0.27;0.68)	90 (0;275)	60 (0;270)	0.58 (0.37;0.72)
Moderate to vigorous activities	795 468;2095)	770 (430;1600)	0.54 (0.32;0.70)	450 (231;795)	413 (238;720)	0.23 (-0.03;0.46)
vigorous activities (min/wk)	140 (0;563)	120 (0;420)	0.59 (0.38;0.75)	320 (120;522)	245 (164;420)	-0.005 (-0.23;0.26)

^a ^Cut-off values for sedentary (<2 MET), light (2–5 MET), moderate (5–8 MET) and heavy (≥ 8 MET) intensity physical activities for adolescents [10].

^b ^Cut-off values for sedentary (<2 MET), light (2–4 MET), moderate (4–6.5 MET) and heavy (≥ 6.5 MET) intensity physical activities for adults [10].

### Validity study

The validity study included 42 adolescents (21 male, 21 female with a mean age of 13.4 ± 1.0 yrs) and 47 young adults (17 male, 41 female with a mean age of 30.1 ± 3.6 yrs). Table 3 shows that the self-reported time spent on sedentary activities was lower compared to the accelerometer. Times spent on all physical activities were higher based on the questionnaire compared to the accelerometer.

**Table 3:** Physical activities and sedentary time by self-report and accelerometry for the validity study (median (25^th^–75^th ^percentile))

	Adolescents (n = 42)	Adults (n = 47)
*AQuAA*		
- AQuAA score (MET*min/wk)	8464 (5146;8465)	6938 (4170;11045)
- Sedentary activities (min/wk)	3000 (2415;3600)	3045 (2455;3610)
- Light activities (min/wk)	810 (600;1335)	1050 (545;1744)
- Moderate activities (min/wk)	565 (348;1019)	160 (25;360)
- Vigorous activities (min/wk)	35 (0;155)	210 (150;480)
*Accelerometer*		
- counts/min	430 (339;510)	355 (299;432)
- Sedentary activities (min/wk)	4838 (4602;5076)	5307 (4956;5458)
- Light activities (min/wk)	910 (764;1165)	711 (616;888)
- Moderate activities (min/wk)	43 (11;66)	108 (63;186)
- Vigorous activities (min/wk)	1 (0;4)	1 (0;26)

#### Hypothesis 1 and 2

The correlations between the AQuAA and Actigraph are presented in Table 4. In adolescents, the Spearman correlation coefficients between the time spent on sedentary, light, moderate and vigorous activities according to the AQuAA and the comparable accelerometer data were 0.23, 0.11, -0.21 and 0.21, respectively (not significant). For adults, the Spearman correlation coefficients between the time spent on sedentary, light, moderate and vigorous activities according to the AQuAA and the accelerometer were 0.15, 0.07, -0.06 and 0.12, respectively (not significant).

**Table 4:** Spearman rank-correlation coefficients between the AQuAA and the Actigraph accelerometer for adolescents and adults.

	Adolescents (n = 42)	Adults (n = 47)
Sedentary activities	0.23	0.15
Light activities	0.11	0.07
Moderate activities	-0.21	-0.06
Vigorous activities	0.21	0.12
Moderate to vigorous activities	-0.23	0.02
AQuAA score^a^	0.13	-0.16

^a ^The AQuAA score includes all activities above 2 MET in MET*min/wk

Comparison of the AQuAA with the Actigraph accelerometer suggests that both adolescents and adults underestimated the time spent on sedentary activities, and overestimated their levels of activities. In adolescents the median difference between accelerometer and questionnaire was 1868 minutes (25;75^th ^percentile 1109;2242) for sedentary activities; -90 (-451;418) for light activities, -540 (-987;-240) minutes for moderate activities; and -35 (-153;0) for vigorous activities. In adults, the median difference between accelerometer and questionnaire was 2216 minutes (25;75^th ^percentile 1579;2729) for sedentary activities,-502 (-1051;121) minutes for light activities, -108 (-286;84) minutes for moderate activities and -314 (-440;-148) for vigorous activities.

## Discussion

A new, self-administered measure of adolescents' and adults' physical and sedentary activities was developed, the **A**ctivity **Qu**estionnaire for **A**dults and **A**dolescents (AQuAA). In adolescents the test-retest reliability for time spent on light intensity physical activities was poor (ICC = 0.30). For the other scores, test-retest reliability was fair to moderate, with ICCs ranging from 0.44 to 0.59. In adults the test-retest reliability was fair to moderate for the time spent on sedentary, light and moderate intensity activities (ICCs ranging from 0.49 to 0.60), but poor for the total AQuAA score and time spent on vigorous activities.

The correlations between the AQuAA and Actigraph were generally low and nonsignificant. Thus, construct validity between the AQuAA and Actigraph could not be confirmed. Absolute comparison of the AQuAA with an accelerometer shows that both adolescents and adults report higher levels of activity than as registered by the Actigraph. Few studies evaluated absolute validity using accelerometers as comparison measure. Most of these studies indicated higher estimates of physical activity by self-reports both in youth as well as in adults, particularly regarding vigorous intensity activities [17]. This finding is in agreement with our results and suggests that self-reports may not be acceptable measures when absolute amount of physical activity needs to be assessed.

The fair to moderate test-retest reliability may be due to true differences in activity patterns because at both administrations the questionnaire recalled a different week. About half of the participants mentioned that their activity level was more or less active than usual. Time spent on all activities was consistently lower in the second week. A plausible explanation for this finding is the fact that for some subjects the second week included one bank holiday. Another explanation may be that participants were more aware of the time spent in different activities due to filling out the questionnaire the first time. Questionnaires recalling a habitual week will most likely have higher test-retest reliability.

The lack of significant correlation coefficients between AQuAA and Actigraph suggests, assuming that the Actigraph can be considered as the criterion standard, that both our age groups had problems with accurately recalling the duration and intensity of the activities they performed in the past seven days compared to accelerometry. Sallis and Saelens [17] summarized validity correlations for seven physical activity measures evaluated in adults. Validity correlations for summary measures of adults' habitual or global physical activity were generally low, ranging from 0.14 to 0.36. A review in children and adolescents found validity correlations between self-reports and accelerometers ranging from -0.26 and 0.40 [18]. In the light of these review findings, it should be concluded that the AQuAA questionnaire showed a lack of overall construct validity with the Actigraph. This finding is comparable to the study of Hagstromer et al [20]. They found low and non-significant correlations between the International Physical Activity Questionnaire (IPAQ) – which was slightly modified for use in adolescents – and the Actigraph in adolescents of approximately the same age. Kurtze et al [21] found low correlations between the IPAQ and hours moderate and vigorous physical activity using accelerometry in young adults.

The correlation between the AQuAA and accelerometer was higher for the time spent on vigorous activities compared to moderate and light intensity activities. In general higher validity for self-report of vigorous activities is observed [17]. This is likely due to the structured and habitual nature of organized sports that make up most of the time spent on vigorous activities. The planned nature of these activities makes them easier to remember.

The low correlation between the AQuAA and the Actigraph was disappointing. A possible explanation for the low correlation between the AQuAA and the accelerometer is the design of the study. The accelerometers were worn for two weeks and after these two weeks the AQuAA was administered. Possibly, some participants answered the questionnaire over the past two instead of one week.

Another explanation for the low construct validity is the choice of the cut-off points for categorizing activities as light, moderate, or vigorous intensity PA as suggested by Freedson [16]. The low levels of moderate (43 and 108 min/wk, respectively) and vigorous (1 min/wk) intensity physical activity both in adolescents and adults suggest that these cut-off points may have been incorrect. The lack of consensus about accelerometer cut points hampers not only comparison of accelerometry with PA questionnaires but also comparability between studies.

Evaluation of the criterion validity of physical activity questionnaires is problematic because there is no gold standard available. Neither of the methods used to assess PA in this study is a gold standard, and both have their shortcomings. The Actigraph accelerometer underestimates certain activities such as weight-bearing activities, cycling and swimming. In the Netherlands cycling is a common activity both in adolescents and adults. Adolescents aged 15–17 cycle the most, on average over six kilometres per day, adults about 2 km per day [22]. Therefore, accelerometry may be less suitable as a comparison instrument in countries where cycling is highly prevalent such as the Netherlands.

According to Sallis et al. [23] comparison of a questionnaire with an accelerometer will consistently underpredict the ability of a questionnaire to accurately assess total PA. High correlations will therefore not be found since both measures concern only a component of the behaviour. Numerous limitations of self-reports have been discussed such as social desirability and recall bias. Furthermore, respondents and investigators must share understanding of ambiguous terms such as physical activity, moderate intensity and leisure time [17]. In the present study the correlations between the AQuAA and Actigraph were low and nonsignificant. This might be interpreted as if both measures assess different PA constructs. The AQuAA provides qualitative information about the different activities performed in the last seven days, while the Actigraph provides objective estimates of the duration and intensity of activities.

This study has a number of limitations. In the reliability study questionnaires were administered in different settings, which could have influenced the results. Also the two tests did not recall the same week. In both the reliability as well as the validity study our focus was on adolescents and young adults. Additional research in larger groups of adolescents and adults with a large age range is needed, where the accelerometer is worn only during the same seven days as the AQuAA is questioning about. To gain more insight in the validity of the AQuAA we recommend further validity research comparing the AQuAA with other comparison instruments such as the newer instrument combining accelerometry and heart rate monitoring. We also recommend methods such as cognitive interviewing to determine whether respondents share researchers' understandings of the concepts and questions used in the questionnaire.

## Conclusion

In summary, we found this self-administered PA questionnaire to be moderately reproducible while the validity compared to an accelerometer was poor. The questionnaire combines information on intensity, duration, and frequency of both physical as well as sedentary activities of both adults as adolescents in one instrument. The agreement in time spent on sedentary, light, moderate and vigorous activities was small but comparable between adults and adolescents.

## Competing interests

The authors declare that they have no competing interests.

## Authors' contributions

MC, SM, AS, and WvM participated in the design of the study and contributed intellectual input into the main ideas of this paper. MC performed the statistical analysis and drafted the manuscript. SM and MvZ coordinated the data-collection. All authors read and approved the final manuscript.

## Pre-publication history

The pre-publication history for this paper can be accessed here:

http://www.biomedcentral.com/1471-2288/9/58/prepub

## Supplementary Material

Additional file 1 **Activity Questionnaire for Adults and Adolescents (AQuAA)**. This files shows the questionnaire. Click here for file
