# Supplementary material for: Reliability and validity of the Activity Questionnaire for Adults and Adolescents (AQuAA)
Source: BMC Med Res Methodol. 2009 Aug 10;9:58. doi: 10.1186/1471-2288-9-58 (PMC3224705; doi:10.1186/1471-2288-9-58)
Supplement: Additional file 1 — Activity Questionnaire for Adults and Adolescents (AQuAA). This files shows the questionnaire. [file 1471-2288-9-58-S1.doc]

**Appendix 1. Activity Questionnaire for Adults and Adolescents (AQuAA)**

Think about the past week (seven days). Please indicate how many days in this week you performed the following activities, how much time on average you were engaged in this per day, and (if applicable) how strenuous this activity was for you?

| **1. COMMUTING ACTIVITIES** | | | |
| --- | --- | --- | --- |
|  | **Days**  **per week** | **Average time**  **per *day*** | **Effort** |
| Walking to/from work and school | …day(s) | …hour(s), …minutes | slow/moderate/fast |
| **Bicycling to/from work and school** | …day(s) | …hour(s), …minutes | slow/moderate/fast |
| **Public transport, car or motor scooter to/from work and school** | …day(s) | …hour(s), …minutes |  |
| **Not applicable** | ….. |  |  |
| **2.** **activity at work AND school** Walking during lunch breaks should be filled in part 4: leisure time activities | | | |
|  | | **Days per week** | **Average time per *day*** |
| **Light work**  E.g. sitting/standing with some walking, e.g. a desk job, following classes^b^, making coffee^a^. | | …day(s) | …hour(s), …minutes |
| **Moderate work**  E.g. work with regular walking (the stairs), walking carrying light objects, cleaning, physical education, delivering the newspapers^b^. | | …day(s) | …hour(s), …minutes |
| Intense work  E.g. walking (the stairs) carrying heavy objects like a heavy bag/schoolbag^b^. | | …day(s) | …hour(s), …minutes |
| **Not applicable** | | ….. |  |
| **3. HOUSEHOLD ACTIVITIES** (in and around the house) | | | |
|  | | **Days per week** | **Average time per *day*** |
| **Light household work**  E.g. Cooking, washing dishes, making the bed, child care at home^a^ | | …day(s) | …hour(s), …minutes |
| **Moderate household work**  E.g. vacuuming, walking/carrying light objects, sweeping. | | …day(s) | …hour(s), …minutes |
| **Intense household work**  E.g. walking with heavy shopping bags | | …day(s) | …hour(s), …minutes |
| **Not applicable** | | ….. |  |

| **4. LEISURE TIME ACTIVITIES**  **Commuting activities to/from work or school excluded. Active sports should be filled in at part 6.** | | | |
| --- | --- | --- | --- |
|  | **Days**  **per week** | **Average time**  **per *day*** | **Effort** |
| **Walking**  E.g. to/from the supermarket, walking during lunch break, walking the dog. | …day(s) | …hour(s), …minutes | slow/moderate/fast |
| **Bicycling**  E.g. to/from supermarket, sports club, cinema. | …day(s) | …hour(s), …minutes | slow/moderate/fast |
| **Gardening/Odd jobs**  E.g. mowing the lawn (non-electric), painting walls, carpentry | …day(s) | …hour(s), …minutes | light/moderate/intense |
| **Not applicable** | | ….. |  |
| **5. SEDENTARY LEISURE TIME ACTIVITIES** | | | |
|  | | **Days per week** | **Average time per *day*** |
| **Watching television** | | …day(s) | …hour(s), …minutes |
| **Using the computer**  E.g. surfing the Internet at home, playing computer games | | …day(s) | …hour(s), …minutes |
| **Reading** | | …day(s) | …hour(s), …minutes |
| **Other sedentary activities**  E.g. talking with friends, board games, sitting in the car | | …day(s) | …hour(s), …minutes |
| **Not applicable** | | ….. |  |
| **6. ACTIVE SPORTS**  **Write down the sports you performed the last week (maximum of 3 sports).**  **Start with the most active sport.** E.g. tennis, fitness, skating, swimming and dancing. | | | |
|  | **Days**  **per week** | **Average time**  **per *day*** | **Effort** |
| **1. …………………………………………** | …day(s) | …hour(s), …minutes | light/moderate/intense |
| **2. …………………………………………** | …day(s) | …hour(s), …minutes | light/moderate/intense |
| **3. …………………………………………** | …day(s) | …hour(s), …minutes | light/moderate/intense |
| **Not applicable** | ….. |  |  |

^a^ example for adults only

^b^ example for adolescents only
